# Information Foraging for Perceptual Decisions

**DOI:** 10.1037/xhp0000299

**Published:** 2016-11-07

**Authors:** Casimir J. H. Ludwig, David R. Evens

**Affiliations:** 1 School of Experimental Psychology and Bristol Vision Institute, University of Bristol

**Keywords:** attention, computational models, decision making, eye movements, information search

## Abstract

We tested an information foraging framework to characterize the mechanisms that drive active (visual) sampling behavior in decision problems that involve multiple sources of information. Experiments 1 through 3 involved participants making an absolute judgment about the direction of motion of a single random dot motion pattern. In Experiment 4, participants made a relative comparison between 2 motion patterns that could only be sampled sequentially. Our results show that: (a) Information (about noisy motion information) grows to an asymptotic level that depends on the quality of the information source; (b) The limited growth is attributable to unequal weighting of the incoming sensory evidence, with early samples being weighted more heavily; (c) Little information is lost once a new source of information is being sampled; and (d) The point at which the observer switches from 1 source to another is governed by online monitoring of his or her degree of (un)certainty about the sampled source. These findings demonstrate that the sampling strategy in perceptual decision-making is under some direct control by ongoing cognitive processing. More specifically, participants are able to track a measure of (un)certainty and use this information to guide their sampling behavior.

Decision making typically involves searching for and sampling information from both external (Hertwig & Erev, 2009; Hills & Hertwig, 2010; Krajbich, Armel, & Rangel, 2010) and internal environments (e.g., memory; Hills, Jones, & Todd, 2012; Stewart, Chater, & Brown, 2006). The way information is sampled can influence the outcome of the decision in perceptual tasks (Glaholt & Reingold, 2009, 2011; Russo & Leclerc, 1994; Shimojo, Simion, Shimojo, & Scheier, 2003), value-based judgments (Krajbich, Armel, & Rangel, 2010; Krajbich, Hare, Bartling, Morishima, & Fehr, 2015; Krajbich & Rangel, 2011; Noguchi & Stewart, 2014) and in risky gambles (Hills & Hertwig, 2010). Most theory development across these different domains of decision making has focused on the overarching decision process: How do humans use the acquired information in order to come to a decision? In this article, we focus on the mechanisms that govern sampling behavior itself. We can think of sampling behavior as a lower level of decision making about what information to acquire, from what source and at what point in time. To obtain a handle on these sampling mechanisms, we focus on decision making in the perceptual domain.

Most studies of perceptual decision making deal with situations in which there is just a single source of information, from which evidence in favor of the various decision alternatives is acquired in parallel. Take, for instance, the classic perceptual decision making task of motion direction discrimination (Britten, Shadlen, Newsome, & Movshon, 1992; Gold & Shadlen, 2000; Huk & Shadlen, 2005; Newsome, Britten, & Movshon, 1989; Palmer, Huk, & Shadlen, 2005; Roitman & Shadlen, 2002; Shadlen & Newsome, 2001). The observer views a single pattern of moving dots. A subset of the dots moves coherently in one of two signal directions (e.g., left vs. right); the remaining dots move in random directions. Therefore, a single source of information provides all the evidence to support a choice between two alternatives: for example, the more rightward motion energy in the stimulus, the less evidence in favor of a leftward choice and vice versa.

Decision making in this type of paradigm is frequently modeled with an evidence accumulation or sequential sampling model. For instance, in the drift diffusion model a decision variable that signals the net evidence in favor of two choice options drifts toward one of two decision boundaries that represent the available options (Ratcliff, 1978; Ratcliff & Rouder, 1998; Ratcliff & Smith, 2004; Smith & Ratcliff, 2004). The drift rate is noisy, possibly because of noise in the information itself and because of noise internal to the decision mechanism. The noise allows the model to account for variability in both choice latency and probability. In an accumulator model the different decision alternatives are represented by different “accumulators” that integrate the evidence in favor of the option they represent (Brown & Heathcote, 2005, 2008; Smith & Vickers, 1988; Usher & McClelland, 2001). Decision making is then governed by a parallel race to a decision boundary. As in the diffusion model, the accumulators are subject to various forms of internal noise, which allows the model to account for variability in choice and reaction time (RT).

Many ecological decision problems involve visually sampling information from a number of sources, which together inform some course of action. For example, when choosing where to place our foot on a rocky path, we visually sample a variety of candidate locations with gaze in the process of committing to a particular step (Patla, 1997). In a supermarket, we might visually compare different packages of cereal before selecting one to put in our trolley. In these situations, the evidence used for decision making has to be gathered in sequence, through selection by overt attentional mechanisms.

In recent years, there has been a great deal of interest in the relationship between gaze behavior and decision making (Glaholt & Reingold, 2009; Krajbich et al., 2010, 2015; Krajbich & Rangel, 2011; Noguchi & Stewart, 2014; Russo & Leclerc, 1994; Shimojo et al., 2003). Specifically, it has been shown that the option fixated for longer and fixated last is more likely to be chosen (Glaholt & Reingold, 2009; Krajbich et al., 2010; Krajbich & Rangel, 2011; Shimojo et al., 2003). These influences of gaze behavior on overall choice have been modeled with a diffusion model in which the drift rate is fixation dependent (Krajbich et al., 2010; Krajbich & Rangel, 2011). In this modification of the drift diffusion model, the drift rate, in addition to an overall bias toward one of the options, is momentarily biased toward for the option currently fixated. As a result, the decision variable drifts toward the boundary representing the currently fixated option and that option is more likely to be chosen.

This model accounts for overall choice preferences and response times. However, the model does not (attempt to) account for the sampling behavior itself. Instead, it assumes that the fixation times are effectively randomly controlled: they are not influenced by the cognitive processing during a fixation. This theoretical stance is reminiscent of *indirect* control models of eye movement behavior in reading and visual search (Hooge & Erkelens, 1996; Vaughan, 1982; Vitu, O’Regan, Inhoff, & Topolski, 1995). However, the currently dominant models in these domains (Engbert, Nuthmann, Richter, & Kliegl, 2005; Henderson & Ferreira, 1990; Nuthmann, Smith, Engbert, Henderson, 2010; Reichle, Pollatsek, Fisher, & Rayner, 1998; Reichle, Pollatsek, & Rayner, 2012) all allow for at least some degree of *direct* control. That is, the duration of an individual fixation may be influenced by the current cognitive processing duration that fixation—an assumption sometimes referred to as the “eye-mind link.”

Although some studies of value-based decision making carefully control the value, or desirability, of the choice options, it is difficult to know what information is being extracted when the participant is fixating an option. As such, it is difficult to characterize the mechanisms that govern sampling behavior. In that regard perceptual decisions offer a better opportunity to control what information needs to be gathered and the quality of that information.

## Information Foraging in Perceptual Decision Making

**Figure 1 fig1:**
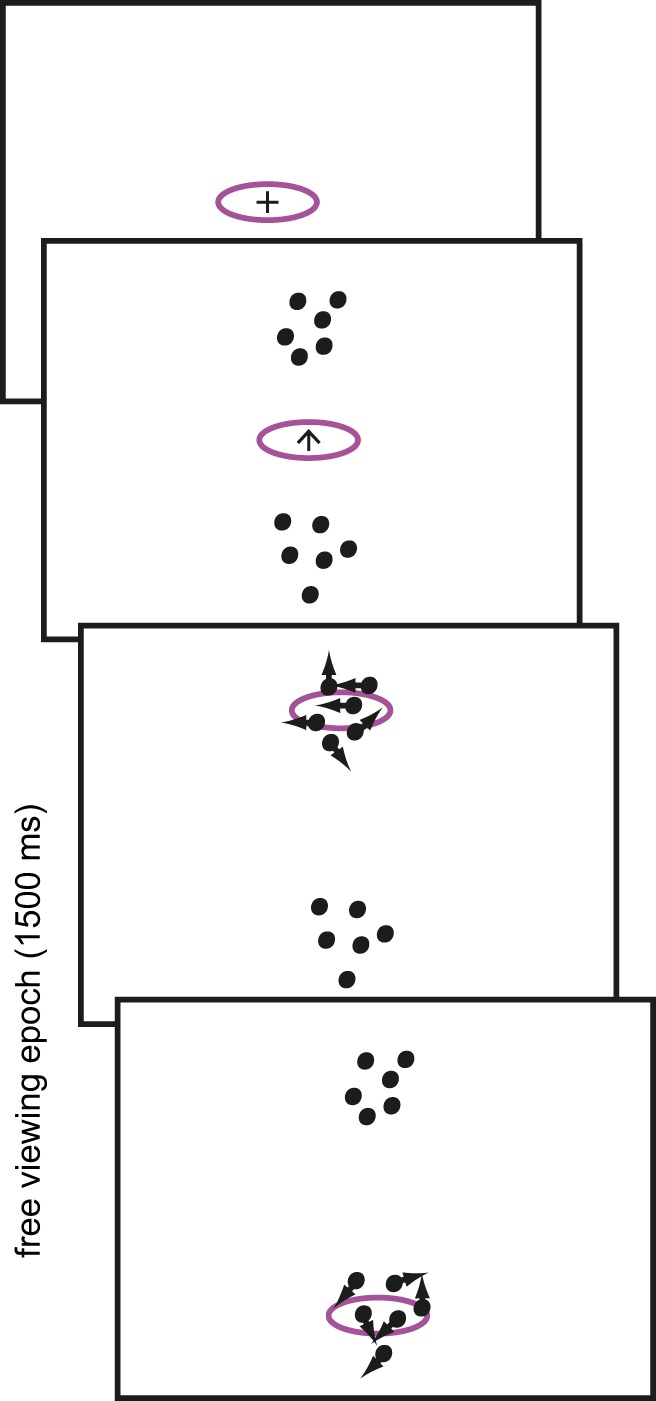
Comparative motion discrimination paradigm used by Cassey et al. (2013) and in Experiment 4 of the current study. Motion information is delivered in a gaze-contingent manner so that a pattern only starts moving once it is fixated. The observer is cued which pattern to fixate first, to ensure that the first fixation is equally often on the top and bottom patterns. Once the eyes have left the central fixation region, the observer has 1.5 s to sample both patterns to come to a perceptual decision. There are no further constraints on fixation behavior. See the online article for the color version of this figure.

In a recent study (Cassey, Evens, Bogacz, Marshall, & Ludwig, 2013), we extended the classic random dot motion task to the situation of interest here in which multiple sources of information have to be sampled serially in order to make a perceptual decision. Figure 1 illustrates the paradigm. Cassey et al. (2013) asked participants to make a comparative motion direction judgment. Observers were instructed with a cue at central fixation which of the two patterns to fixate first. Gaze position was monitored online and motion information was only delivered once a pattern was fixated. From the moment the eyes left the central fixation, observers had 1.5 seconds to sample the two patterns. The task was to indicate which of the patterns moved relatively more clockwise (through the short angle). For example, in Figure 1 the coherent motion of the top pattern is toward 9 o’clock and the motion of the bottom pattern is toward 7 o’clock. In this case then, the top pattern moves relatively more clockwise. If the directions of motion had been, say, 11 and 1 o’clock, the 1 o’clock pattern should be classed as more clockwise. The quality of the two patterns is easily varied by changing the motion coherence.[Fig-anchor fig1]

This comparative motion direction discrimination paradigm incorporates some of the critical features that are ecologically relevant and (mostly) quite distinct from the “standard” perceptual decision making paradigm reviewed above. (a) There are multiple sources of information, which can only be sampled one at a time; (b) The quality of information provided by different sources may vary, but (c) the quality (coherence) is independent from the task-relevant variable that needs to be extracted (direction); (d) The quality of a source is only known once it has been or is being sampled; (e) The task-relevant variable that needs to be extracted from a source is continuous, rather than a discrete classification; and (f) The information sampled from multiple sources has to be put together in order to form some overall, categorical judgment about the “state” of the environment.

We think of the problem loosely as a “foraging” task in a patchy environment (Charnov, 1976; McNamara & Houston, 1985; Pirolli & Card, 1999; Stephens, 2008) The objective is to gather as much information as possible from multiple patches in a limited period of time. Information may be defined as a measure of uncertainty reduction (MacKay, 2003): more information means that we are more certain about the value of some variable. In the context of value-based decision making, sampling an option may make the observer gradually more certain about the utility of that option, or more certain about the difference in utility between that option and some alternative. In the context of the comparative motion discrimination task described above, sampling a pattern may make the observer gradually more certain about the motion direction of that pattern. In this article, we adopt a loose definition of information as the degree of certainty around the task-specific variable that is important for decision making.

**Figure 2 fig2:**
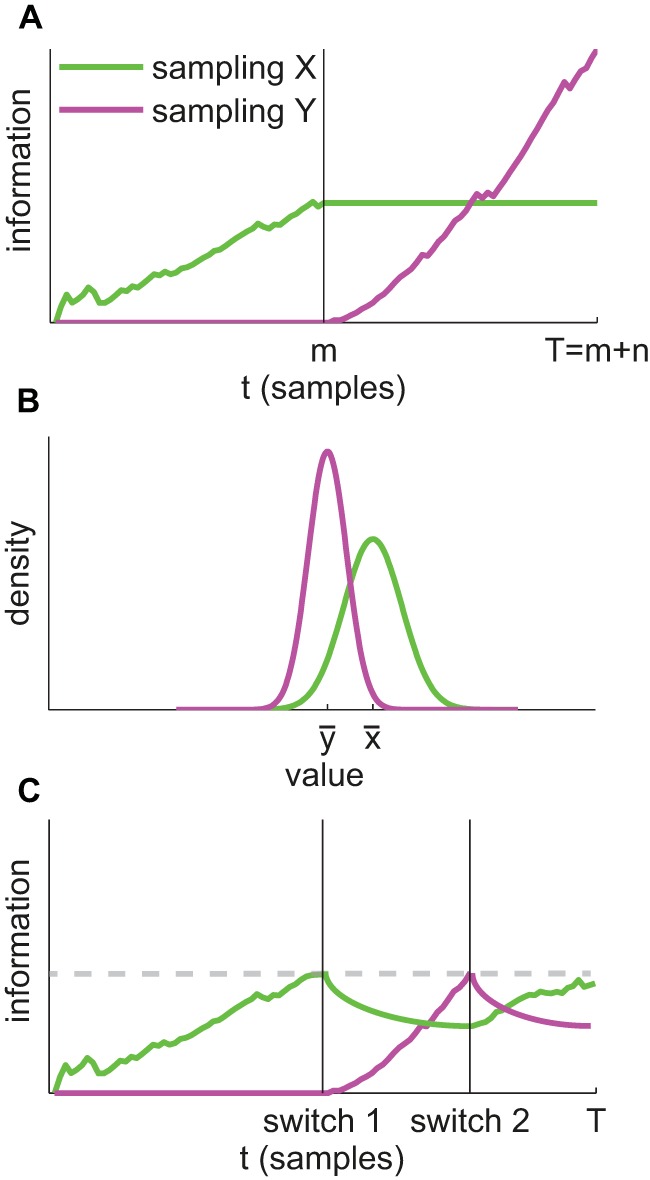
Information foraging account. There are two sources of information which are sampled serially. A – Accumulation of information for both sources. The observer first samples variable *X* and midway through the available time switches to variable *Y*. When a source (i.e., *Y*) has not been sampled yet, the information about that source is 0. *X* provides lower quality information than *Y*, which translates into a different rate at which information is accrued. B – At the end of the sampling time *T*, the information accumulated for both options sets the width of a set of internal response distributions that represent the values of both options. For the sake of illustration, variable X has been given a higher mean value. C – Sampling controlled by placing a threshold (gray dashed horizontal line) on the information accumulated. In addition, previously sampled information may degrade when a new source of information is being sampled, shown by the gradual decrease for *X* while sampling *Y* (and then for *Y* while sampling *X* for the second time). Once the information criterion for the second source has been reached, the observer has time left to switch back to the first source and acquire more information from *X*. See the online article for the color version of this figure.

Figure 2 sketches the information foraging account. We assume there are two sources of information, only one of which can be sampled at a time. A comparison of the task-relevant variable (generically indicated as ‘value’) is performed at the end of the total sampling period. Panel A shows that the observer first samples source *X*. After *m* samples, (s)he switches to source *Y* and takes *n* samples from that source. The total sampling time is given by *T* = *m* + *n* and in this example *m* = *n* because the observer switches exactly halfway. Suppose source *X* is of lower quality than source *Y* and the samples drawn from *X* are more variable. Panel A illustrates how information grows as the samples come in and demonstrates that the rate of information accumulation depends on the quality of the source (i.e., lower slope for *X*). After switching to *Y*, the information accumulated from *X* is maintained in working memory (Romo & Salinas, 2003).[Fig-anchor fig2]

At the end of the available sampling time, the observer has a representation of the values of both *X* and *Y* and the (un)certainty of these representations is determined by the total accumulated information for each source. In this example, at time *T* the observer has accrued more information for *Y* than for *X*, which translates into a smaller spread of the corresponding distribution in panel B. The accuracy with which X can be discriminated from Y clearly depends on the amount of information accumulated from both sources.

Suppose the observer has some way of monitoring the amount of information accumulated (i.e., certainty around the estimated value of a source). In that case, (s)he might try to obtain equally good estimates for both sources of information. One way to achieve this goal is simply to impose some criterion on the information. The gray dashed horizontal line in Figure 2C shows a hypothetical criterion. It is clear that were such a criterion in place, it would take longer to reach it when sampling *X* compared to sampling *Y*. In this way, the observer would spend more time sampling a low quality source of information compared to a high quality source of information.

It is also possible that the working memory representation of previously sampled information is not perfect, unlike the perfect maintenance of information shown in Figure 2A. Panel C shows how the representation of the previously sampled source may degrade over time once the observer has switched to a different source. Such degradation of information may arise as a result of passive decay (forgetting) or interference in working memory (Barrouillet, Portrat, Vergauwe, Diependaele, & Camos, 2011; Lewandowsky & Oberauer, 2009; Oberauer & Lewandowsky, 2008). In the face of such degradation, it may be adaptive to switch back to the first source once the criterion amount of information has been accumulated for the second source. Indeed, Cassey et al. (2013) hypothesized that such imperfect memory was one source of inefficiency in human observers and the reason why participants regularly switched back to a previously sampled source in their task. Note that in Figure 2C the total sampling time for *X* is much greater than for *Y* and that this difference occurs through (a) a more prolonged first sampling epoch, and (b) a subsequent return to that source. In this way the model can account for adaptation of the total sampling time to the quality of available information, as observed by Cassey et al. (2013).

In some way, the framework sketched in Figure 2 resembles standard accumulator models that are used to account for RTs and choice data in simple decision making tasks (Brown & Heathcote, 2005, 2008; Smith & Vickers, 1988; Usher & McClelland, 2001). Indeed, as in a standard accumulator model, the rate of rise of an “accumulator” (say, the green line) is determined by the quality of the evidence provided. However, there are some important differences.

Most importantly, the information foraging framework is an attempt to represent an overall more challenging situation that involves multiple sources of evidence, which can only be sampled serially. This situation calls for switching between sources of information and (working) memory demands. The accumulators shown in Figure 2 represent the (un)certainty around a continuous value estimated from a source of information. Good evidence means that a criterion is reached more quickly. However, unlike in a standard accumulator model, this larger drift does not mean that the decision alternative represented by the accumulator is more likely to be chosen in the end. Indeed, in the example shown in Figure 2 option *X* has the higher value, but provides worse evidence. In other words, the traces shown in Figure 2, are similarly influenced by the quality of sensory evidence as in standard accumulator models, but they do not represent the evidence *in favor of that option*.

That said, it could be argued that each individual sampling “episode” is like a basic perceptual decision about a single random dot motion pattern, which may be described by some rise-to-threshold mechanism. However, note that this decision involves identifying the continuous value of a pattern, rather than a discrete classification into a limited number of response categories. Standard models of (perceptual) decision making rely on a limited number of decision alternatives, represented by separate decision boundaries (Smith & Ratcliff, 2004) or accumulators (Brown & Heathcote, 2008; Smith & Vickers, 1988). Such models are not well suited to describe decisions on a continuous scale. Extensions of these models so that they involves some continuous decision “field” (Ludwig, Mildinhall, & Gilchrist, 2007; Smith, 2016; Wilimzig, Schneider, & Schoener, 2006) may be able to account for the sampling behavior in our particular paradigm and we will highlight this possibility in the General Discussion.

The foraging framework outlined above has several components that need to be tested and empirically constrained. First, information—in the form of certainty around the parameter of interest—grows with sampling time. Second, information stored in working memory from the previously sampled source(s) may degrade with time. Third, the point at which a source is left, is determined by monitoring the amount of information accrued *online*. In Experiment 1, we aimed to characterize the growth in information with time. In Experiment 2, we characterized the growth in information in the presence of a memory representation of a previously sampled information source. In addition, this experiment was designed to identify any loss of previously sampled information after switching to a new source. In Experiment 3, we aimed to identify the origin of limited information growth observed in Experiments 1 and 2. Finally, Experiment 4 tested whether the decision to leave a source of information is driven by online monitoring of the information accrued.

## Experiment 1

The foraging framework assumes that certainty grows with sampling time. There are numerous demonstrations of psychophysical discrimination ability improving with time in a wide variety of domains (for a review, see Watson, 1986), including random dot motion direction discrimination (Gold & Shadlen, 2003; Huk & Shadlen, 2005; Kiani, Hanks, & Shadlen, 2008). However, typically accuracy is measured as a threshold for discrimination performance in a forced choice paradigm. In these paradigms, the behavioral decision may be formed by evaluating the relative evidence for a small number of discrete options from a small number of channels (e.g., left vs. right). Here we are concerned with estimating and signaling a more continuous variable, by asking participants to report the direction a random dot motion pattern is moving in. The questions we address here are *how* information grows with time under these conditions and in what way this growth depends on the quality of the information.

This experiment was performed during the larger study by Cassey et al. (2013). Eight participants in that study performed a single session of absolute motion direction judgments, in which we assessed the accuracy of their motion direction judgments for just a single pattern presented for a variable amount of time. Cassey et al. (2013) only used and reported the data from two (of 13) conditions: low and high coherence patterns viewed for ∼750 ms. Here we report the full data set from this experiment as a way to describe how the accuracy of the direction estimates changes over time.

### Method

#### Participants

Eight participants took part in the full study, which involved attending six ∼1-hr sessions on different days. The fourth of these sessions was devoted to measuring the accuracy of their single pattern direction identification.

Ethical clearance for this and all subsequent experiments was obtained from the local Faculty of Science Human Research Ethics Committee. The experiments were conducted in accordance with the ethical guidelines of the British Psychological Society (which are in line with those of the APA). All participants provided written informed consent and were fully debriefed.

#### Materials

Stimuli were created using the PsychToolbox 3.0.8 (Brainard, 1997; Kleiner, Brainard, & Pelli, 2007; Pelli, 1997) in Matlab (The MathWorks Ltd.). They were presented on a 21″ Viewsonic G225fB monitor with 1024 × 768 resolution at 85 Hz. The viewing distance was ∼57 cm, with the head stabilized through a chin and forehead rest. The stimulus was viewed through a hot-plated mirror that is part of an eye tracker, but eye movements were not monitored in this session.

One RDK pattern consisted of 100 light or dark gray dots (squares of side length 3 pixels ≈ 7′) which moved within a circular aperture of radius 4° on a midgray background, giving a mean dot density of 2 dots°s^−2^. The dots moved according to a ‘Brownian Motion’ algorithm (Pilly & Seitz, 2009). That is, on each frame, a subset of dots were chosen as signal dots and translated in the signal direction. The signal direction was chosen randomly for each trial from the interval [0..359], with all signal dots being displaced in that direction. The direction of the noise dots was sampled from the same uniform distribution, with the direction drawn independently for each noise dot and each frame. All dots moved at the same speed at 6°s^−1^, being translated ∼4′ on each frame. RDK animations were produced independently for each trial for each participant.

#### Procedure

A single RDK was shown in the center of the screen for one of six durations: {71, 129, 247, 506, 653, 1000} ms. The coherence of the pattern was 12% (“low”) or 24% (“high”). To identify an upper limit of direction identification performance, we included one additional condition in which a 99% coherence pattern (“super high”) was shown for 1000 ms. Each of the 13 conditions was repeated 55 times, for a total of 715 trials, performed over one hour with three breaks.

Participants simply viewed the RDK and then indicated their estimate of its net motion direction using an on-screen dial positioned with a mouse (see also Figure 4). The initial position of the dial was set randomly so that there was no correlation with the actual motion direction. Participants had unlimited time to position the dial and when they were satisfied with the indicated direction they pressed the mouse button. A green arrow would then display the true motion direction alongside the reported direction.

There are two forms of error in this absolute direction judgment task: systematic error (bias) and variable error. From the perspective of our foraging account, the latter is of particular interest. That is, the variable that participants are trying to estimate is the (mean) direction of the pattern. As the pattern is sampled for longer, the certainty around that estimate is expected to increase. This increase in certainty around the mean is quantified by the variable error. For each direction judgment, we computed the (smallest) angular difference with respect to the true direction of the probed pattern. Across trials, the mean of this distribution of this angular error corresponds to the bias. The spread of this distribution corresponds to the variable error.

### Results

**Figure 3 fig3:**
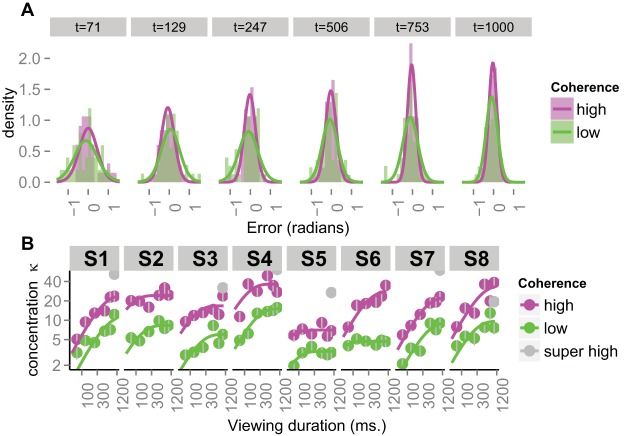
Information growth with sampling time in Experiment 1. A – Error distributions for one participant (S1). The errors are shown in radians. The solid curves are fits of a Von Mises circular density. B – Growth in the Von Mises concentration parameter with sampling time for individual participants. The solid curves are fits of a “speed-accuracy” growth curve (see text for details). Also shown in gray are the concentration estimates from the 99% coherence condition. Where these symbols are not visible (S2 and S6 in particular), it is because they lie beyond the upper limit of the ordinate. Error bars are standard errors. Double (natural) log coordinates are used to facilitate visual inspection of the low and high coherence data within the same panels. See the online article for the color version of this figure.

Figure 3A shows the distributions of errors for low and high coherence RDKs for the different viewing times for one participant. We excluded trials in which the error fell outside the interval ± π/2 (i.e., outside a ± 90° semicircle around the true direction of motion). These error distributions were fit with a Von Mises circular density, which is characterized by two parameters: location μ and concentration κ. The concentration parameter is a reciprocal measure of the spread of the distribution—it is comparable with the precision (reciprocal variance) of a Gaussian distribution. Visual inspection of the full histograms (i.e., including all the trials) suggested that there were occasional minor secondary peaks around ± π; that is, the reported direction was *opposite* to the true direction. We do not have an explanation for this finding. One possibility is that participants may sometimes respond to the orientation of the spatiotemporal pattern (Adelson & Bergen, 1985) or to motion “streaks” (Apthorp & Alais, 2009).[Fig-anchor fig3]

When using the full circular range we found that the fitted Von Mises concentration parameter would have to decrease to accommodate these outliers, often at the expense of capturing a well-defined central peak. As the number of these outliers was relatively small and variable across conditions and participants, including these trials decreased the reliability of our concentration estimates at the individual level. Qualitatively the results were similar when the full circular range was used. We also explored fitting mixtures of Von Mises distributions (e.g., Zhang & Luck, 2008), but again found that estimates at the individual participant level were less reliable, with the overall pattern preserved. Given these observations, we report the analyses conducted on the truncated distributions. Over all conditions and participants 9% of the trials were discarded because of errors that were too large. Such errors were not evenly distributed across the conditions, with more rejected trials for short viewing times compared with long viewing times.

Several features of the distributions in Figure 3A are worth noting. First, all distributions are closely centered around zero. In other words, there was very little bias and on average this participant was accurate in the direction judgments (as, indeed, were the others). Second, the low coherence distributions are wider than the high coherence distributions. This result confirms that the variation in coherence was sufficient to induce a variation in the precision with which the direction could be estimated. Third, the distributions are reasonably well characterized by Von Mises densities, particularly for the longer viewing times. At early viewing times, the distributions are more uniform and the fits do not capture the tails very well. Nevertheless, the Von Mises concentration parameter is a reasonable index of the spread of the distribution. Fourth, for both coherence levels, the distributions become narrower with increasing viewing times. That is, the direction judgments are becoming more precise with an increase in sampling time.

Figure 3B illustrates the increase in concentration with sampling time for each individual participant in the study. The data in panel A correspond to Participant 1 (S1) in panel B. The low and high coherence concentration estimates are fit with “speed-accuracy” growth functions of the form: $\kappa \left(t\right)=\lambda \left(1-e^{-\frac{t}{\tau }}\right)$ for *t* > 0 (McElree & Carrasco, 1999; Ludwig & Davies, 2011).

It is clear that for almost all participants, with exception of S5 perhaps, there is strong growth in the concentration with time. In addition, for all participants, there is a clear separation between the low and high coherence concentration estimates: the high coherence patterns allow for much more accurate direction judgments. Both the low and high coherence data grow to asymptotic levels (given by λ in the equation above; the asymptotes are more clearly visible on linear coordinates). Importantly, the asymptotic levels appears differ for the two coherence levels. That is, it is not the case that the functions appear to converge to a common asymptote. We return to this issue below in Experiments 2 and 3. Finally, the concentration of the direction estimates for the super high coherence pattern was generally much larger than the upper limit of the high coherence precision (in some cases off the scale; see Figure legend).

A more detailed statistical analysis of the parameters of the growth functions (asymptotes and time constants: λ and τ respectively) will be deferred until the results for Experiment 2 have been presented.

## Experiment 2

In Experiment 2 we again probe the growth of information, but this time under conditions in which the direction of a previously sampled pattern has to be maintained in working memory. In addition, we assessed the loss of information once the observer has switched to a new source of information. It is clear that shifting the focus of attention to new material has the capacity to interfere with the maintenance of material stored in memory (e.g., Logie, Zucco, Baddeley, 1990). Moreover, temporal decay and interference are common mechanisms used to account for forgetting (Barrouillet et al., 2011; Oberauer & Kliegl, 2001; Oberauer & Lewandowsky, 2008). However, it remains to be seen whether such degradation of previously sampled material occurs in sensory information foraging.

In this experiment two RDK patterns were viewed in sequence, with participants actively switching from one pattern to another. Participants were probed to report the direction of either the first or the second viewed RDK. The idea here is that when the first pattern (RDK1) is probed, the viewing time of the second pattern (RDK2) is effectively a delay interval. Moreover, processing of the motion of RDK2 may interfere with the memory representation of the RDK1 direction. When the second pattern is probed, we again identify the growth in information over time, but under conditions in which that information was processed with a concurrent memory load (RDK1 direction).

### Method

#### Participants

Eight new participants were tested in five sessions, run on different days, with each session lasting approximately one hour. In the first session, participants performed four blocks of 96 trials. In the remaining four sessions, they performed five blocks of 96 trials, for a total of 24 blocks (2304 trials/participant). Participants were paid £50 upon completion of all five sessions.

#### Materials

The stimuli were created in mostly the same way as in Experiment 1. In this experiment the RDKs consisted of white dots on a black background. The RDKs were positioned ∼5.8° above and below the center of the screen. The position of one eye (typically the right) was recorded at 1000 Hz using an Eyelink 2000 video-based eye-tracker (SR Research Ltd.). The viewing distance was ∼57 cm. To direct gaze to the appropriate locations, we used a small, light gray fixation cross (a ‘+’ sign, with each leg measuring 0.5° × 0.1°). Eye movements were tracked to control the stimulus presentation in a gaze-contingent manner, but were not analyzed.

#### Procedure

**Figure 4 fig4:**
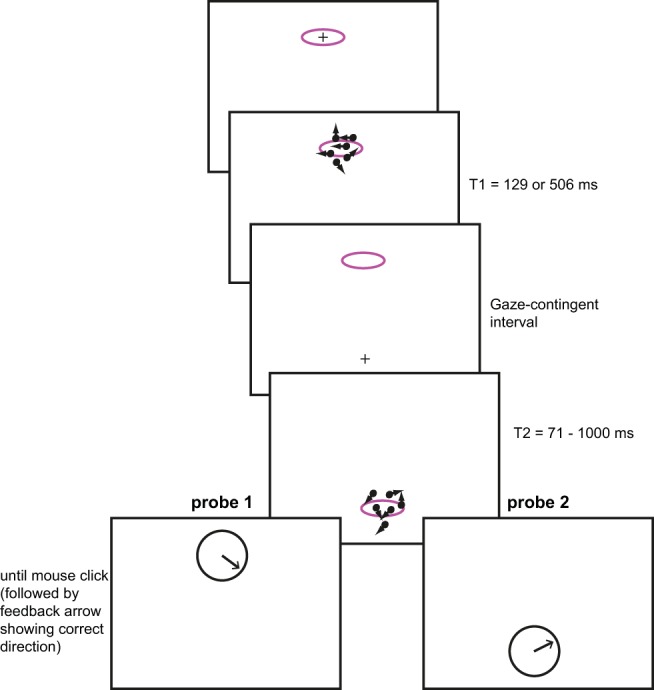
Characterizing information growth and loss in Experiment 2. When we probe the participant to report RDK2, the variation in *T*_2_ allows us to track the growth of information with time. When we probe the participant to report RDK1, then *T*_2_ acts as a delay interval. We use this delay to track the loss of information after switching to a new source. See the online article for the color version of this figure.

Figure 4 illustrates the paradigm used in this experiment. Participants started by fixating a target in the either the top or bottom pattern location. Once accurate fixation was achieved, a foreperiod of 800 ms was followed with presentation of RDK1 in this location. The pattern could have either a low or a high coherence (12 or 24%, respectively; we omitted the 99% coherence condition). RDK1 was presented for either 129 or 506 ms (*T*_1_ ∈ {129, 506}), after which it disappeared and a fixation point appeared in the location of RDK2. The activation of RDK2 was gaze-contingent: the pattern was presented as soon as the vertical gaze position crossed an invisible, horizontal boundary at a distance of 1.8° from the center of the screen (in the same visual field as RDK2). The second pattern was presented for a variable amount of time: *T*_1_∈ {71,129,247,506,753,1000}. Gaze contingency was introduced in this experiment to ensure that the demand for active switching was as similar as possible as the paradigm used by Cassey et al. (2013) and in Experiment 4 here.[Fig-anchor fig4]

After the offset of RDK2, a dial appeared with the arrow pointing to a randomly set direction. The location of the dial cued the participant to report the global motion direction of either RDK1 or RDK2 (probe 1 or probe 2, respectively in Figure 4). The participant set the direction of the dial using the mouse cursor and clicked the mouse when (s)he was satisfied with the direction set. At that point the true motion direction was shown for 500 ms (in the form of a green arrow, drawn together with the light gray arrow set by the participant).

There were 24 conditions in which we probed RDK1, defined by combining: the coherence of the first pattern (low/high), *T*_1_ (two levels) and *T*_2_ (six levels). Each of these conditions was repeated twice in a block. There were 12 conditions in which RDK2 was probed, through combining the coherence of the second pattern (low/high) with *T*_2_ (six levels). In these conditions, both the coherence and the presentation time of RDK1 were chosen randomly. Each of these 12 conditions was repeated 4 times in a block, so that RDK1 and RDK2 were probed equally often. The combination of number of conditions and repetitions per condition, created 96 trials in a block; these trials were randomly intermixed.

### Results

**Figure 5 fig5:**
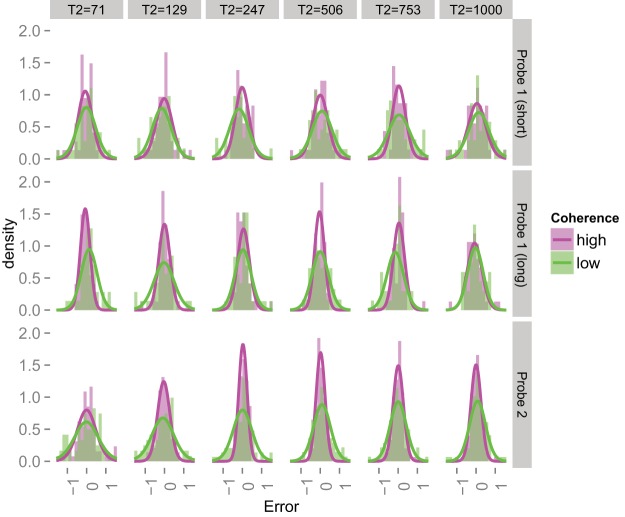
Direction judgment error distributions for one participant from Experiment 2. Solid lines are fits of a Von Mises density. See the online article for the color version of this figure.

For each participant, Experiment 2 generated 36 error distributions (24 RDK1 conditions; 12 RDK2 conditions). Figure 5 shows all 36 distributions for one participant. In the top two rows, we probed the participant to report the direction of RDK1. The viewing time of RDK2 (along the columns) here acts as an increasing delay interval. The bottom row shows the errors when RDK2 was probed; this row is directly comparable to Figure 3A. As in Experiment 1, we rejected trials in which the indicated direction fell in the wrong “half” of the circular dial. Across all participants and conditions, 11% of the trials were rejected for this reason. The remaining errors were fit with circular Von Mises densities.[Fig-anchor fig5]

Once again, the distributions are centered around zero and the low coherence distributions are generally wider than the high coherence distributions. If we take the top two rows, there appears to be no consistent change in the shape of the distributions along the columns, perhaps with exception of the low coherence distributions in the first row (RDK1 probed and viewed for 129 ms.). These distributions become wider as the delay interval (*T*2) increases. This is the kind of change we might expect to see in the case of working memory decay or interference (i.e., the participant becomes less precise the longer the delay interval).

In the bottom row we characterize the growth in precision, now with the added complication of having to keep the direction of RDK1 in mind while estimating the direction of RDK2. For both coherence levels the distributions appear to get narrower, at least for the short viewing times, again suggesting that the concentration of direction estimates grows with sampling time.

**Figure 6 fig6:**
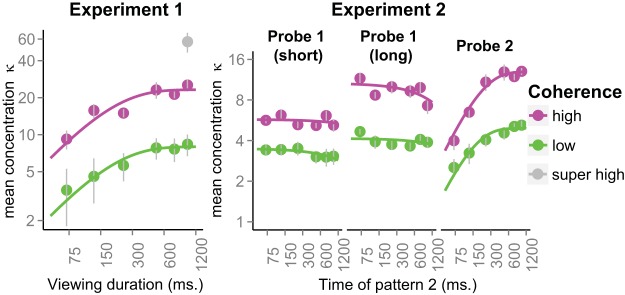
Concentration of the direction estimates in Experiments 1 and 2, averaged across participants. The growth curves (left-most and right-most panels) are characterized by the “speed-accuracy” function, similar to those shown in Figure 3B. When RDK1 is probed (middle two panels), we fit the data with an exponential decay function. Error bars are within-subject SEMs (Morey, 2008), calculated for each experiment separately. See the online article for the color version of this figure.

From these error distributions we estimated 36 concentration parameters for each participant. Figure 6 (right three panels) shows these parameters averaged across participants. The right-most panel shows the mean concentration of RDK2 errors as a function of how long that pattern was viewed. The growth in accuracy is immediately obvious and it is well characterized by a speed-accuracy function of the same form as shown in Figure 3B. Again, both functions grow to an asymptote and those asymptotes depend on the coherence level. For comparison, the left-hand panel shows the growth curves from Experiment 1, averaged across participants for that experiment. The nature of information growth appears very similar to Experiment 2, except that the asymptotic concentration estimates are higher in Experiment 1. We return to this issue below with a statistical assessment.[Fig-anchor fig6]

The middle two panels of Figure 6 show the concentration of direction judgments of RDK1, after an interval during which RDK2 is sampled. These data were fit with a simple exponential decay function of the type: $\kappa \left(t\right)=\lambda e^{-\frac{t}{\tau }}$, for *t* > 0 (shown by the solid lines). There appears to be some loss of information in some conditions (low coherence, probe 1-short; high coherence, probe 1-long).

To assess whether any information loss is reliable, we examined the estimated τ parameters of the exponential decay function. If we take the natural logarithm of the concentration estimates, λ and τ may be estimated with the following linear regression equation: $\ln\;\kappa \left(t\right)=\lambda -\frac{1}{\tau }t$. Therefore, if $\frac{1}{\tau }$ is positive, the function has a negative slope; if $\frac{1}{\tau }$ is negative, the function has a positive slope.

We compared the slopes from all four conditions against zero (no loss, no growth). We used Bayes Factors in order to quantify the evidence in favor of a difference from zero, but also to be able to quantify evidence in favor of the null hypothesis (Rouder, Speckman, Sun, Morey, & Iverson, 2009; Wagenmakers, 2007). The Bayes Factor compares two models: a null model of no difference and an alternative model that predicts there is a difference from zero. The Bayes Factor is effectively a marginal likelihood ratio, averaged over a prior distribution of effect sizes. In line with Rouder et al. (2009), We adopted a scaled Cauchy prior on the effect size with a medium scale of $\sqrt{\left(2\right)}/2$. This prior places most of its mass on small-to-medium effects. Bayes Factors were computed using the BayesFactor package in R and the ‘ttestBF’ function (Morey, Rouder & Jamil, 2014).

Although the slopes were generally close to zero, all but two of the 32 slope estimates (eight participants times four conditions) were negative. The Bayes Factors in favor of the alternative were: 1.36, 3.70, 3.22, and 1.97 (high-short, high-long, low-short, low-long conditions, respectively). Bayes Factors between 1/3 and 3 are generally considered ambiguous (Raftery, 1995). Bayes Factors between 3 and 10 would provide some evidence in favor of the alternative model. According to these rules of thumb, although the slopes are consistently negative, we only have some evidence in favor of a nonzero slope in the low-short and high-long conditions. Note that these inferences are relatively independent of our assumptions about the scale of the prior: adopting a wider prior on the effect sizes (a value of 1 on the Cauchy *r* scale parameter) changed the Bayes Factors very little. In summary, we have some evidence for information loss, but the evidence is relatively weak and the effects are clearly small (see also comment in the General Discussion).

Next we turn to the growth in information with sampling time. Here we focus on two questions. First, do the rate of growth and asymptotic concentration depend on the coherence of the pattern? Second, do the rate of growth and asymptotic concentration change in the presence of a concurrent memory load (i.e., having to hold the direction of a previously sampled pattern in mind in Experiment 2)? Note that these analyses are post hoc and exploratory: we had not fully planned Experiment 2 at the time of running Experiment 1.

To address these questions, we analyzed the growth data from Experiments 1 and 2 together in a Bayes Factor mixed-design ANOVA (Rouder, Morey, Speckman, & Province, 2012), with coherence as a within-subject factor, experiment as a between-subjects factor, and participant as a random factor. This analysis compares a succession of more complex models against a null model that only has a subject-specific intercept. To address the questions about growth rate, the dependent variable was the fitted τ parameter of the growth curves. To address the questions about variations in asymptotic concentration, the dependent variable was the fitted λ parameter of the same functions.

The logic of this approach is rooted in the idea of model selection (Burnham & Anderson, 2002). That is, we compare models with main effects (and/or interactions) to a simple null model that contains only a subject-specific intercept. If adding a predictor helps account for variance in the dependent variable, then a model that contains this predictor should perform better relative to the null model, even when penalized for its greater complexity and flexibility. Likewise, we can compare different models that contain different main effects and/or interactions with each other in a similar way. The Bayes Factor tells us how much our prior beliefs in a pair of models (e.g., null vs. main effects) should shift in light of the data obtained.

Table 1 lists the Bayes Factors for the three main effects models and the full main effects + interaction model, against the null model. Values greater than one indicate support for the alternative model; values below one indicate support for the null model. For the growth rate, τ, we have no evidence against the null at all. The Bayes Factors for the four models are all below one. For the two most complex models (simple main effects model: Experiment + Coherence; main effects and interaction model: Experiment × Coherence) there is actually some weak evidence *in favor* of the null. In other words, we have no evidence that τ varies with coherence or working memory load.[Table-anchor tbl1]

For asymptotic precision, λ, the strongest model is one that contains both main effects of experiment and coherence, and their interaction. Of the two main effects, including coherence on its own clearly accounts for substantial variation in the data. Adding experiment to this model improves its fit: the Bayes Factor for the Experiment + Coherence model, relative to Coherence on its own is just under 6 in favor of the more complex model (divide the Bayes Factors for Models 3 and 2). This improvement stems from the overall higher concentration without a concurrent memory load in Experiment 1. Adding the interaction gives a Bayes Factor of just over 4 relative to the main effects model, so there is some relatively weak evidence that the effect of coherence depends on the experiment. Although it appears that we have some evidence that a working memory load affects the upper limit of accuracy that can be achieved, we revisit this issue in Experiment 3.

## Experiment 3

In both Experiments 1 and 2, we observed limited growth in the accuracy of direction judgments with time, with a strong dependence of the asymptote on coherence. Experiment 3 served two purposes. First, we attempted to distinguish between several alternative explanations for the limited growth. Second, this experiment served as an additional check on the higher asymptotes observed without a working memory load in Experiment 1.

With regard to the limited growth of information, we consider a number of possible explanations. First, an asymptote would arise in the presence of a stimulus-independent source of noise that cannot be “integrated out” by sampling the stimulus for longer. One example of such a noise source would be motor noise in the setting of the dial (Green & Swets, 1966; van den Berg, Awh, & Ma, 2014). We can reject this explanation already on the basis that we observe *different* asymptotes for low and high coherence patterns. A stimulus-independent noise source would predict a common asymptote that does not depend on coherence. Indeed, the generally much higher concentration observed in the 99% coherence condition of Experiment 1 (see Figures 3B and 6) also indicates that performance in the low and high coherence conditions was unlikely to be limited by this type of stimulus-independent noise.

Second, a variation on the first hypothesis is that there are other sources of internal noise that *do* depend on the stimulus (i.e., greater internal noise the lower the coherence) and that cannot be integrated out. Low-level observer models of the visual system sometimes include an “induced noise” component: a source of internal noise that scales with the amount of external noise in the stimulus (Burgess & Colborne, 1988; Eckstein, Ahumada, & Watson, 1997; Lu & Dosher, 2008). If this noise source was static over time, then sampling the stimulus for longer would not diminish its influence (Ludwig & Davies, 2011). An example of a static internal source of noise is between-trial noise in the drift rate in evidence accumulation models (Brown & Heathcote, 2005, 2008; Ludwig & Davies, 2011; Ratcliff & Rouder, 1998).

Third, the limited growth may indicate that participants are making their direction judgments based on only a subsample of the sensory evidence. There are several reasons why this inefficiency might have occurred. (a) The sensory mechanisms that signal the instantaneous motion direction to an integrator unit may have a relatively transient impulse response (Adelson & Bergen, 1985). Such mechanisms would respond vigorously to the onset of the RDK pattern and some brief period after that. However, the mechanisms would become increasingly unresponsive to sustained delivery of motion information. In other words, the integrator would, after a brief period, no longer be getting new evidence samples. (b) The sensory mechanisms may be sustained, but participants “make up their mind” after some period of time before the viewing period is over (Ratcliff, 2006). They effectively stop paying attention to the later evidence samples, perhaps in the belief that their direction estimate is already good enough. We will refer to hypotheses (a) and (b) collectively as a ‘primacy gradient,’ because both hypotheses predict that the early evidence samples carry greater weight (c). Integration of sensory evidence may be leaky, that is, subject to decay (Teodorescu & Usher, 2013; Usher & McClelland, 2001). Note that this form of decay is somewhat different from the information loss probed in Experiment 2. Leaky integration implies that earlier evidence samples are effectively replaced by later samples. This mechanism predicts that the later evidence samples carry greater weight compared to the earlier sample that have been forgotten (Bronfman, Brezis, & Usher, 2016).

In this experiment we directly tested this third hypothesis that decisions are based on a subsample of the evidence. If participants do not use all the available information, we aimed to identify whether they give a relatively greater weight to the early information (as predicted by the primacy gradient hypotheses (a) and (b) above) or to the later information (as predicted by the leaky integration hypothesis (c)). Our approach was to perturb the quality of information delivered to the observer briefly, either early or later in time.

**Figure 7 fig7:**
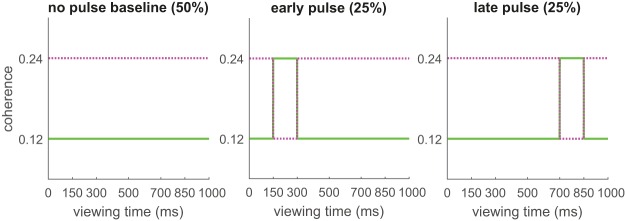
Pulse methodology used in Experiment 3. On pulse trials, the coherence of the RDK is briefly altered: either from low to high (green traces) or high to low (magenta traces). The pulse either comes early or late in the viewing period. See the online article for the color version of this figure.

Figure 7 illustrates the logic of our approach. Participants judged the direction of a single RDK, just as in Experiment 1. The viewing duration was fixed to 1 s (the longest viewing period in Experiments 1 and 2). On half the trials, we briefly altered the coherence of the pattern, with a coherence “pulse.” The magnitude of the pulse was ± 12%, subtracted from a high coherence RDK and added to a low coherence RDK. The choice of pulse magnitude was determined in a pilot experiment to ensure that it would actually affect the accuracy of people’s direction estimates. In the same pilot we verified that the pulses themselves could not be reliably identified. The pulse was delivered either early in the viewing period or toward the end.[Fig-anchor fig7]

The idea here is that when the coherence is low, a brief increase in the coherence should improve accuracy, but only to the extent that participants were sensitive to the sensory evidence delivered in the early or late pulse epoch. Under the primacy gradient hypotheses, participants will be more sensitive to early information compared with later information. Therefore, early (positive) pulses should be more beneficial than late pulses. Under the leaky integration hypothesis, early information decays in favor of later evidence samples. Therefore, a late coherence pulse should be more beneficial than an early pulse.

For high coherence patterns, the effect of the (negative) pulses should be opposite (i.e., a brief decrease in coherence should decrease accuracy). However, the high coherence motion direction can be estimated with high precision in a relatively short period of time anyway. As such, we might expect that an early negative pulse can easily be overcome with (limited) later evidence samples. Likewise, a late pulse may not detract much from an already good direction estimate. On this basis, the positive coherence pulses added to the low coherence patterns are the most diagnostic with regard to the different hypotheses under investigation. Nevertheless, we included the complementary high coherence (negative pulse) condition for two reasons. First, the high coherence, negative pulse condition is used in Experiment 4 to test the prediction that in free sampling conditions, (un)certainty plays a role in governing the switch point from one source to another. Second, inclusion of ‘no pulse’ baseline trials, for both coherence levels, is a reproducibility check on the asymptotic concentration levels that can be achieved in the absence of a working memory load. That is, if the concentration values estimated on these trials match those of Experiment 1, we may be increasingly confident that the overall decrease in concentration in Experiment 2 (i.e., in the presence of a memory load) is real and not just attributable to the use of a different sample of participants.

### Method

#### Participants

Eight new participants were recruited. They took part in a single session that lasted approximately 1 hour. Participants were paid £8 for their help.

#### Materials

The stimuli were created in the same way as in Experiments 1 and 2. Only a single RDK was presented in the center of the screen for 1 s. The RDK consisted of white dots on a black background. Participants viewed the screen through the hot-plated mirror used in eye tracking, with their head constrained with chin and forehead rests. Eye movements were not recorded, but in this way we controlled the viewing distance (at ∼57 cm) and matched the viewing conditions to those in all other experiments reported here. Coherence pulses were delivered either early or late in the viewing period. The pulse duration was ∼150 ms (14 frames at 85 Hz refresh rate). The early pulse started ∼150 ms into the animation (on frame 13); the late pulse started ∼700 ms (on frame 59) into the animation.

#### Procedure

A trial started with an 800 ms fixation cross, which was then replaced by the RDK animation for 1 s. The RDK was followed by the dial and after setting it, participants received feedback as in previous experiments. The next trial would then start automatically after a delay of 750 ms. Participants were told that they could take a break by setting the dial, but withholding the mouse click. In addition, forced breaks were introduced every 80 trials.

The two coherence levels, crossed with pulse presence/absence and pulse location (early/late), gives eight experimental conditions. Each condition was repeated 60 times, for a total of 480 trials. The different conditions were randomly intermixed. On trials in which there was no pulse, a 0% magnitude pulse was allocated to either the early or late epoch. In this way, we ensured that the assessment of the pulse effect for each combination of coherence and pulse location was based on a similar number of trials.

### Results

**Figure 8 fig8:**
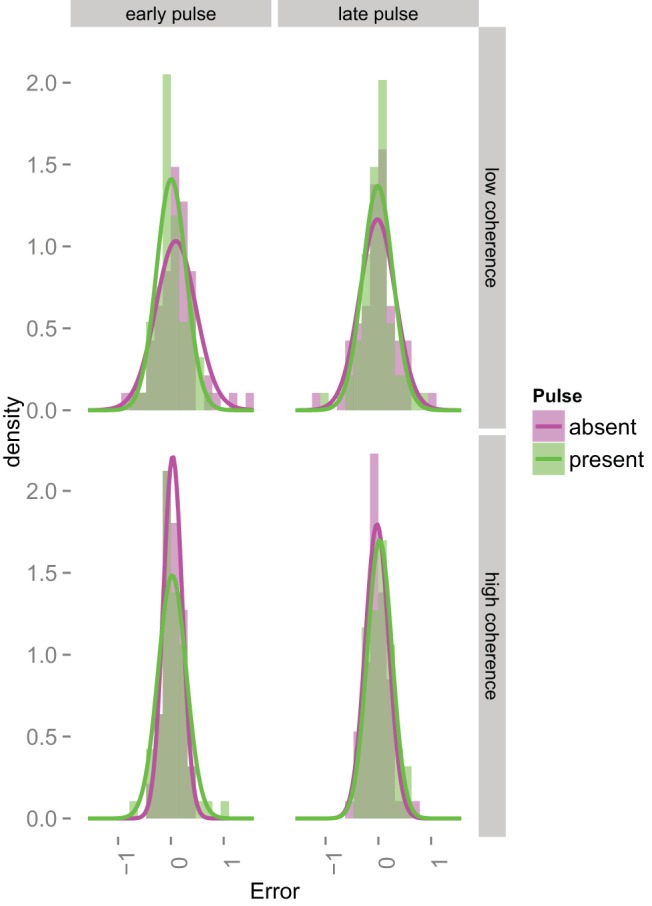
Direction judgment error distributions for one participant from Experiment 3. Solid lines are fits of a Von Mises density. See the online article for the color version of this figure.

Figure 8 shows the distributions of direction errors in all eight conditions for one participant. The different colors in each panel now correspond to the presence or absence of a coherence pulse. On the top row, we show the data from the low coherence conditions. Here we expect a pulse to be beneficial for at least one of the epochs. A benefit of the pulse should manifest itself as a narrower distribution with a higher peak. This effect can be seen quite clearly for both early and late pulses for this participant.[Fig-anchor fig8]

The bottom row shows the high coherence data. We were noncommittal about our expectations here, for reasons given above. If the pulse was going to affect accuracy at all, we would expect it to harm performance. Such an effect would manifest itself as a wider distribution with a lower peak. For this participant, the early pulse appears to have detrimental effect, but the later pulse does not appear to affect performance much at all.

**Figure 9 fig9:**
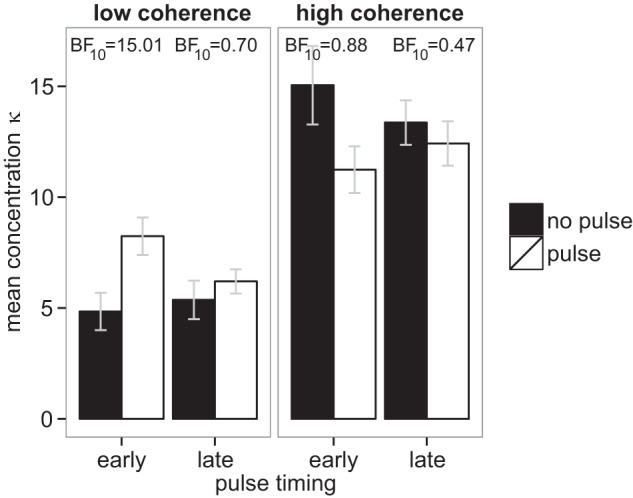
Mean concentration estimates (± within-subject SEMs) for all eight conditions in Experiment 3. The Bayes Factors for each paired comparison quantify the evidence in favor of an effect of the coherence pulse (i.e., values greater than 1 suggest an effect).

Figure 9 shows the concentration estimates from the Von Mises fits, averaged across participants. Note that within each panel, the no pulse trials are indistinguishable and should generate similar concentration estimates. Reassuringly, in both panels, the within-subject error bars for early and late no pulse trials overlap substantially.[Fig-anchor fig9]

The benefit of a positive pulse (left-hand panel) should manifest itself as higher concentration values in the pulse trials. Any decrease in performance from a negative pulse (right-hand panel) should show up as a decrease in the concentration values in the pulse trials. We simply tested for a pulse effect for each combination of coherence and pulse location. The Bayes Factors in favor of the pulse having an effect is given near the top of each panel, above each pair of bars. There is clear evidence in favor of a positive effect of an early pulse for low coherence RDKs. It appears that there is also an effect of the early pulse for high coherence RDKs, but the variability here is greater and the Bayes Factor is ambiguous. When the pulse was delivered late, the evidence is again ambiguous.

Finally, we note that the no pulse concentration values observed in this experiment are more similar to the 1 s concentration estimates obtained in Experiment 2 (Figure 6—right-hand panel) than in Experiment 1 (Figure 6—left-hand panel). This observation qualifies the effect of working memory load on asymptotic concentration values in the comparison of Experiments 1 and 2. It appears that the concentration values in Experiment 1 were elevated, perhaps because this experiment was part of a larger study and participants had already performed three sessions in which they had to make comparative direction judgments between RDKs. As such, when evaluated across Experiments 1 through 3, we have little evidence that a concurrent working memory load influences the integration of sensory evidence.

## Experiment 4

Having characterized the way information grows with time when sampling a source of information, we now turn to the question whether participants are able to monitor the amount of information accumulated *online* and use this to decide when to switch from one information source to the next. The basic prediction is straightforward: when the first source that is sampled is of low quality (high noise, low coherence), the observer should switch later than when that source is of high quality (low noise, high coherence). In addition, when a low quality source is temporarily enhanced with good quality information, the participant should switch earlier compared to when this enhancement is absent. Likewise, when a good quality source is temporarily degraded, participants should delay their switch point. These key predictions were tested in Experiment 4.

In this experiment, we used the comparative motion discrimination paradigm of Cassey et al. (2013) and illustrated in Figure 1. To test the online accrual of information, we embedded an early coherence pulse in the first RDK on half the trials. That is, if the coherence of the first pattern was low, it would be increased briefly, and if the coherence of the first pattern was high, it would be decreased briefly (as in Experiment 3). If the switch point is controlled by monitoring the (un)certainty of the motion direction estimate for that pattern, we would expect the switch point to be delayed for a negative pulse and to be brought forward for a positive pulse.

### Method

#### Participants

Eight participants were tested in three sessions, run on different days with each session lasting approximately one hour. The first session was used to estimate a fixed, angular offset between the two patterns that was to be used in the main experiment. The main experiment was then run in two additional sessions consisting of 6 blocks of 64 trials. Participants were paid £25 upon completion of all three sessions.

#### Materials

The equipment used was the same as that in Experiments 1 through 3. The algorithm to generate the RDK patterns and their spatiotemporal parameters also remained the same. Again, we used two coherence levels of 12% and 24%. In the main experiment, a “standard” direction was chosen randomly from the interval [0..359] and assigned to either the top or bottom pattern. The other pattern then moved in the standard direction ± a fixed angular offset determined by the preliminary measurement of the directional discrimination threshold.

#### Procedure

The first session was dedicated to the measurement of a fixed angular offset to be used in the main experiment between the two patterns. Adapting the offset for each individual participant was important for two reasons. First, in this way all participants performed at approximately a similar level of accuracy. Second, we needed to find an angular offset where the variation in coherence actually mattered. That is, if the offset is really large it may be easy to detect even when the coherence of the RDKs is low.

Participants viewed two RDKs in sequence, presented at the center of the display. The patterns always had the same coherence within a trial, but varied between the low and high coherence levels. Eye movements were not monitored. Each pattern was presented for 600 ms with a ∼750-ms interstimulus interval. Participants responded whether the first or the second pattern moved in a relatively more clockwise direction with a button press. The angular offset between the two patterns in either a clockwise or anticlockwise direction was controlled by an adaptive staircase routine (Watson & Pelli, 1983) to target a performance level of 75% correct. We interleaved separate staircases for the low and high coherence levels, with 80 trials/staircase. Participants were given the opportunity to practice until they felt comfortable with the task (typically between 30 and 60 practice trials). The 160 staircase trials were run with a break halfway. The required directional offset for the high coherence patterns is typically much smaller than that for the low coherence patterns. The offset used in the main experiment was set exactly midway through the low and high offsets (typically, across many such experiments in our lab, the offset averages around 40°).

The main experiment involved the same direction comparison, but now participants were free to sample the two patterns with active gaze, as shown in Figure 1. A fixation point appeared in the center of the display, which was used to check for accurate fixation and recalibration of the eye tracker if necessary. An up or downward pointing arrow replaced the fixation point and two stationary dot patterns appeared ∼5.8° above and below central fixation. The arrow remained visible for ∼330 ms. The offset of the arrow was the cue for the participant to shift gaze to the cued pattern. We cued the first pattern in this way to ensure that the top and bottom patterns were fixated first equally often (otherwise people have a tendency to fixate the top pattern first). From the moment the arrow disappeared, participants had ∼1.5 s to freely sample both patterns.

The activation of a RDK was gaze contingent: if the eyes crossed a vertical distance of 1.8° from the fixation, the pattern in the corresponding visual field was turned ‘on.’ We gave no instructions about the number of switches or the strategy that participants should follow. With a predictable offset of the arrow, participants frequently anticipated its disappearance and shifted their gaze around the time of arrow offset. If the eyes were already on the pattern before the full presentation time of the arrow, the RDK would only move upon arrow offset. We did not exclude these trials.

When the whole sampling period was over, participants had to indicate whether the top or bottom pattern moved relatively clockwise by pressing the left or right button (respectively) on a standard game pad. There was no deadline for the manual response. Auditory feedback was given with 500 and 750 Hz. tones indicating error and correct decisions respectively. The tone was played for 100 ms.

The full design of the main experiment is as follows. The coherence of the first pattern (the one cued at fixation) can be low or high. The coherence of the second pattern was low or high, independently from the first pattern coherence. Either the top or the bottom pattern was cued first with equal probability. The “standard” was allocated to either the top or bottom pattern and the other pattern moved in a direction either clockwise or anticlockwise from the standard. On half the trials a coherence pulse was inserted while the participant sampled the first pattern. The pulse magnitude was ± 12%, subtracted from a high coherence RDK and added to a low coherence RDK. Full factorial combination of these factors results in a block of 64 trials which were randomly intermixed. The intertrial interval was ∼750 ms.

Trials were rejected if participants failed to sample both patterns and if they switched away from the first pattern before the pulse was complete (i.e., fixation durations less than 306 ms). We applied the same duration criterion for no pulse control trials. The proportion of rejected trials varied between 3–12% across all eight participants. Our results did not change qualitatively or statistically without rejecting any trials.

The eye tracker was calibrated using a grid of 9 points at the start of each block of trials. The calibration target was a ‘+’ with each leg measuring 0.5° × 0.1°. The calibration target was also used as the fixation point at the start of each trial.

Saccades and fixations were parsed offline using velocity and acceleration criteria of 30°s^−1^ and 8000°s^−2^. The onset of a fixation on a pattern was determined by the offset of the saccade that shifted gaze onto that pattern. The offset of a fixation was the onset of the saccade that took gaze away from that pattern (typically back to the central region before moving on to the other RDK). The critical outcome measure is the fixation duration on the pattern sampled first. Note, that this period may encompass several fixations within the same pattern. Therefore, we refer to this measure as the *switch* time.

To facilitate comparison with previous work from Cassey et al. (2013), we report three additional variables. The total fixation time on a pattern (including return visits) corresponds to the ‘gaze time’ for that pattern. The gaze time on the first pattern is not independent from the total gaze time on the second pattern, due to the fixed trial duration. We express the gaze time on the first pattern as a proportion of the total gaze time over the trial (i.e., $\frac{GT_{1}}{GT_{1}\;+\;GT_{2}}$). We also examined the number of switches on each trial and we report the proportion of trials in which only one switch occurred. Finally, we checked the overall accuracy of the perceptual decisions.

### Results

Table 2 summarizes the mean (±95% confidence intervals) gaze time (proportion of time spent on the first pattern), proportion of 1-switch trials and perceptual accuracy across the sample of eight participants, separately for the four combinations of two coherence levels. The no pulse control conditions are directly comparable with the data from Cassey et al. (2013, their “unknown” condition) and replicates the results reported by those authors. We highlight some salient findings, although these are not of primary interest here.[Table-anchor tbl2]

First, the overall gaze time on the first pattern (i.e., including refixations) was clearly affected by the coherence of that first pattern, with the low coherence pattern being fixated for longer. It is also notable that more than half the available gaze time was allocated to the first pattern (all proportions are above 0.5), even when the quality of both patterns was the same.

Second, there is a modulation of the gaze time by the coherence of the second pattern. That is, participants spent relatively less time sampling the first pattern when the quality of the second pattern was low. This was the critical effect of Cassey et al. (2013). Note that any effect of the quality of the second pattern has to be mediated through refixation. That is, participants cannot know what the coherence of the second pattern is before they have switched, so on single-switch trials the gaze time on pattern 1 cannot reflect the unknown quality of the second source. The small magnitude of the effect is attributable to the large proportion of single-switch trials.

Third, the majority of trials involved just a single switch to the second pattern and the participant then kept fixating that pattern until the end of the trial. The proportion of such trials is slightly lower when the second coherence is high, similar to Cassey et al. (2013).

Fourth, perceptual accuracy was close to the 75% level targeted with our titration of the angular offset between the two patterns. Participants could clearly do the task, yet it was still challenging. In addition, the variation in coherence mattered. Performance was worst when the coherence of both patterns was low and it was best when the coherence of both patterns was high. The “mixed” conditions fell between these extremes.

**Figure 10 fig10:**
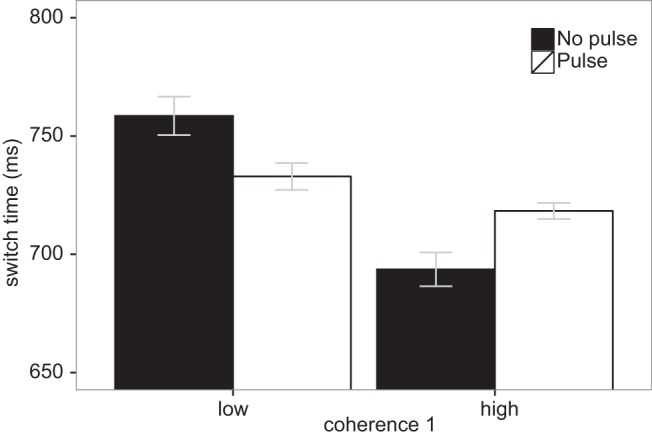
Switch time in the comparative motion direction discrimination task of Experiment 4 (mean of means across eight participants). Error bars are within-subject SEMs.

The critical outcome measure for the present purposes is the switch time. Figure 10 shows the mean switch times in the four conditions created by crossing the coherence of the first patch (low or high) with the presence or absence of the coherence pulse (note that the coherence of the second patch cannot influence the switch time). On no pulse trials, the coherence of the first pattern predictably influenced the switch time, with observers switching later if the coherence was low. However, delivery of a positive pulse in this case brought the switch point forward. Delivery of a negative pulse when the coherence is high delayed the switch point.[Fig-anchor fig10]

Statistically, the primary effect of interest is an interaction between coherence of the first patch and the presence of the pulse. We therefore compared two general linear models. The null model only contains a random effect of participant and fixed effects of the first pattern coherence and presence of a pulse. The “interaction” model contains the same random and fixed effects, as well as the critical interaction. The Bayes Factor quantifies the relative evidence in favor of the interaction model (Rouder et al., 2012). This interaction model was well over 3000 times more likely than the null model. An evidence ratio of this magnitude is considered very strong evidence in favor of the critical interaction (Raftery, 1995).

## General Discussion

The way information is sampled can influence choice behavior in perceptual tasks, value-based decision making and experiential risky choice paradigms (Glaholt & Reingold, 2009, 2011; Hills & Hertwig, 2010; Krajbich et al., 2010, 2015; Krajbich & Rangel, 2011; Noguchi & Stewart, 2014; Russo & Leclerc, 1994; Shimojo et al., 2003). Computational models of decision making typically focus on the overall choice, given that a certain amount of information is available to the decision maker. Our work aims to elucidate the mechanisms that drive the acquisition of information itself. That is, when different sources of information have to be sampled to come to some overall decision, how do observers allocate time to these different sources? How does the time allocation depend on the quality of those information sources? Does the timing of a switch from one source to another tell us something about cognitive processing preceding the switch?

We conducted a series of experiments to test an information foraging framework of the way sensory evidence is sampled in the process of making a perceptual decision. We focused on three key components of this framework: (a) Information about a source grows with time as it is being sampled; (b) Once a new source is being sampled, information about the previously sampled source may degrade; and (c) The point at which the observer switches from one source to another is governed by online monitoring of his or her degree of (un)certainty about the sampled source. We deal with each of these components in turn.

### Information Growth

Experiments 1 and 2 characterized in detail how information—the degree of certainty about motion direction—grows with time. Several features of the results are noteworthy. First, information grows to an asymptote. Second, the asymptotic precision depends on the quality of the source, with the lower quality source having a lower asymptote. Third, the rate of information growth is invariant to changes in coherence and memory load. Fourth, there is little evidence that keeping the direction of a previously sampled pattern in mind influences the extraction of information from a subsequently sampled pattern.

One reasonable question is whether the limited growth in information is somehow inherent in the stimulus. It is not self-evident that for RDK patterns the concentration of directional errors should increase linearly with viewing time. The Appendix describes a set of simple simulations that demonstrates that the concentration of directional errors does indeed increase linearly, and that it reflects the average pattern of information growth *within a trial* (a point we will return to below).

We put forward a number of explanations for the limited growth in information: (a) stimulus-independent static noise (e.g., motor noise); (b) induced (stimulus-dependent), static internal noise; (c) a primacy gradient, where early evidence samples are weighted more heavily than later evidence samples; and (d) leaky integration, where early evidence samples are replaced by newer information. Experiment 3 demonstrates that the growth of accuracy is limited at least partly because not all evidence samples are used equally. That is, early samples are more influential when giving a direction estimate than later samples. These findings are most consistent with the primacy gradient hypothesis (c). The primacy gradient may stem from transience in the impulse response of the motion-sensitive sensory mechanisms, participants deciding on the direction before the viewing period is over, or a combination of both.

### Information Loss

We probed the loss of information in Experiment 2, where participants had to view two patterns in sequence and were asked to report the direction of either the first or the second pattern. When they were probed for the first pattern, the viewing time of the second pattern acted as a (filled) delay interval in which new information from a different source was extracted. On the whole, our measured time constants of information loss were large (for the sample fits shown in Figure 6 the time constants ranged between 3 and 18 s). As such, the amount of information lost even in the largest, 1-s delay period was relatively small.

It could be argued that the delay interval is actually much longer than simply the viewing time of pattern 2. There is a brief interval between the offset of the motion of the first pattern and the onset of the motion of the second pattern, attributable to the instructed saccade from one pattern location to another. In addition, once the second pattern has been shown, there is a RT associated with setting the dial and submitting the response. To a first approximation, however, including these additional delays would simply shift all the data points rightward along the abscissa, increasing the time constants to even larger values.

Two further issues are worth noting. In the absence of new information, some loss of information seems all but inevitable. That is, it is unlikely that people’s direction estimates would become *more accurate* in the absence of new information (although some sequential sampling models assume that decision making is served by sampling from a memory representation; e.g., Smith & Ratcliff, 2009). Therefore, at best one might expect no loss of information (zero slope in Figure 6). A zero slope is achieved by having a very large time constant, but even if every participant had such large time constants, the distribution of slopes ($-\frac{1}{\tau }$ in our regression analysis) would still be negative. As such, it is fair to say that the slope analysis in this experiment is biased against 0.

In addition, it may be argued that the concurrent memory load here is rather minimal. People do not need to maintain a visual working memory representation of the whole, temporally extended pattern they viewed first. They just need to encode that pattern with a single direction estimate (e.g., a visual or verbal representation of a “clock face”). It is possible that the working memory load here effectively corresponds to just one unit or “chunk” (Luck & Vogel, 1997). Such a low load may be relatively easy to maintain, even in the face of processing new visual information.

On the whole then, it is fair to say that the gradual loss of information from working memory was minimal. As such, we have little evidence that return visits in a free sampling paradigm (Experiment 4 and Cassey et al., 2013) to previously sampled information sources are driven by a need to “refresh” previously gathered information. It is possible that these revisits form some kind of verification step (Russo & Leclerc, 1994; Glaholt & Reingold, 2011) or that they occur on trials in which the observer switched before a good estimate of the first source was acquired, on the basis that the overall time is limited.

### Online Monitoring of Information Accrual

A critical hypothesis of the information foraging framework is that when participants have to sample multiple sources of information, the point at which they leave a source is governed by applying some criterion amount of certainty about the task-relevant variable. We tested this hypothesis in Experiment 4, in which we modified the comparative direction discrimination task of Cassey et al. (2013). This paradigm has all the key features of an information foraging task, as outlined in the Introduction. We see this paradigm as a bridge between classic perceptual decision making tasks (e.g., left-right random dot motion discrimination; Gold & Shadlen, 2001), value-based preferences (Krajbich et al., 2010; Krajbich & Rangel, 2011) and risky choice situations that involve active sampling of pay-off distributions (Camilleri & Newell, 2013; Hertwig & Erev, 2009).

This experiment provides two contributions. First, the no pulse data replicated the results reported by Cassey et al. (2013). Second, the pulse manipulation tested whether the switch was driven by online monitoring of the information extracted. The results were clear-cut: the timing of the first switch was strongly influenced by altering the quality of information even just briefly during the fixation (see Figure 10).

These data provide strong evidence against *any* model that assumes that the switch point is not controlled in an online manner by the incoming evidence. For example, indirect control models assume that fixation durations are effectively random (Hooge & Erkelens, 1996; Vaughan, 1982; Vitu et al., 1995), and this assumption is a core feature of the fixation-dependent drift diffusion model used to account for value-based decision making (Krajbich et al., 2010; Krajbich & Rangel, 2011). This form of indirect control can already be rejected based on the no pulse data in Experiment 4: participants switched earlier from a high quality source of information compared to a low quality source. Nevertheless, that effect may be accounted for without necessarily assuming online monitoring of the information accrued. Only ever two coherence levels are used in our comparative direction discrimination task. It is possible that participants pick up on this feature and that they learn to classify the first pattern as low or high coherence early on in the trial. This early classification may be used to set a temporal deadline or switch point for that particular trial. Participants may then monitor some internal timing mechanism (Engbert et al., 2005; Ivry & Hazeltine, 1995; Ivry & Spencer, 2004; Nuthmann et al., 2010; Trukenbrod & Engbert, 2014) to this deadline.

Models in which a temporal deadline is set based on early evidence cannot account for the influence of the pulse, unless they assume that the early evidence is computed in a window of ∼300 ms (over this window the average coherence in the positive and negative pulse conditions was equal). Of course, given limitless flexibility in the window over which the timer or deadline is set, it becomes difficult to distinguish such a model from the online monitoring hypothesis. However, online monitoring of the accrued information appears a less post hoc and more natural explanation of the switch time results.

### Information Foraging in Perceptual Decision Making (Revisited)

With the new empirical findings in hand, we now return to the information foraging framework sketched in Figure 2. Suppose for the moment that the growth curves we have measured (see Figure 6) are representative of the information accrual *during an individual trial*, as illustrated in Figure 2. Clearly it is possible to impose some criterion on the degree of certainty and, in line with the foraging framework, it would take longer to reach that criterion for the low coherence pattern compared to the high coherence pattern. In that sense, the foraging framework is qualitatively consistent with the growth curves we have measured.

However, if we push this model further, the asymptotic growth curves pose a problem. That is, suppose we placed a criterion on, say, a concentration value of 10 (imagine a horizontal line at κ_*c*_ = 10 in Figure 6—left and right panels). It is clear that this criterion will never be reached for a low coherence pattern. Therefore, a common criterion would be constrained by the upper limit of accuracy attainable for the poorest quality information source (e.g., a criterion at κ_*c*_ = 4). This criterion would be exceeded extremely swiftly for a better quality source, even though for that source there would still be plenty of room for further information growth.

An apparently logical alternative would be to impose a criterion that is set relative to the maximum accuracy attainable for a source (e.g., κ_*c*_ = *c*λ, where 0 < *c* < 1 is the relative criterion, and λ is the asymptotic concentration that depends on the quality of the source). However, if accept our finding that the time constant of information growth is the same for low and high coherence patterns, it follows that the time at which this relative criterion is reached will not depend on coherence. This prediction is clearly inconsistent with our switch time results (see Figure 10).

Another alternative, one that is at least superficially more closely related to classic models of animal foraging (Charnov, 1976; Stephens, 2008), is that there is a criterion on the *rate* of information growth. This would make intuitive sense: there is no point staying in a patch when you are no longer gaining very much information from that patch (and, presumably, more information could be gained from a different patch). This mechanism would involve monitoring the slope of the tangent of the growth curve and switching when this slope drops below some criterion value (formally: a criterion on the derivative of the growth curves). However, given equal time constants and differences in asymptotic accuracy, it actually turns out that this slope criterion is exceeded *more quickly* for the low coherence source, again in contrast to the switch time data.^1^

However, we would caution against interpreting our empirical growth curves as directly *equivalent* to the (average) information accrual experienced in real-time during a sampling epoch on any one individual trial in the free sampling paradigm. First, these curves were derived under conditions in which the viewing time was limited, but the subsequent response time was not. The pattern may have been presented for, say, ∼500 ms, but the participants may have taken several seconds to set the dial. Although no new information is presented during this interval, it is possible that the deliberation afforded by this intervening period distorts whatever degree of certainty existed *at 500 ms in real time*. For instance, participants may continue to sample evidence from a memory representation of the pattern (Pleskac & Busemeyer, 2010) and use this to alter their representation of the motion direction.

Second, we have quantified the degree of certainty as the concentration of a Von Mises density fit to the distribution of errors *across* a number of trials. To map this on to what happens *within* an individual trial requires at least two assumptions: (a) The concentration of errors across trials at time *t* represents the average precision with which the mean direction is estimated from the samples observed up until *t* within individual trials; (b) The metric that participants use to track their (un)certainty is closely related to our estimate of their (un)certainty in the form of the concentration of a Von Mises distribution.

With regard to the first assumption, the Appendix outlines the logic of our approach and confirms the validity of this logic through simulation. This simulation demonstrates that the concentration of errors across trials is a good approximation of the average precision around the estimated mean direction within trials. With regard to the second assumption, it is clearly not necessarily the case that the actual metric of information used by participants in real time matches the one we have estimated in our experiments.

In this regard, the idea that an individual sampling episode is like a perceptual decision, but one that involves estimating a continuous value, is worth revisiting. As stated earlier, standard models of (perceptual) decision making, such as the drift diffusion model (Smith & Ratcliff, 2004) or accumulator models (Brown & Heathcote, 2008; Smith & Vickers, 1988) that explicitly represent a small number of discrete decision alternatives are not well suited to this more continuous estimation problem. However, one can think of the decision as the parallel accumulation of evidence in favor of a large number of different directions, for example, as a 1D decision field with many accumulators representing different motion directions with relatively fine granularity (Ludwig et al., 2007; Wilimzig et al., 2006). In a similar vein, Smith (2016) has extended the drift diffusion model to continuous report tasks on a circular scale, such as those frequently used in the visual working memory literature (e.g., Zhang & Luck, 2008).

It is possible that such models may be used to account for the sampling behavior seen in our particular task involving direction judgments of random dot motion patterns. However, the generality of such mechanisms to other decision problems between options specified by continuous values (e.g., value-based judgments and risky choices in decisions from experience; Camilleri & Newell, 2013; Hertwig & Erev, 2009; Krajbich et al., 2010; Krajbich & Rangel, 2011; Lejarraga, Hertwig, & Gonzalez, 2012), remains to be seen. The fundamental claim made in this article is that tracking (un)certainty is online is a useful generic mechanism for governing sampling behavior in a wide variety of decision problems. Evidence accumulation in a continuous decision field is one way to achieve this implicitly, because dynamic noise in the accumulation process is integrated out gradually (Ludwig & Davies, 2011), resulting in more precise responses as time goes by. However, more explicit measures of uncertainty may also be used. For example, participants may use confidence as a proxy measure of their (un)certainty, where confidence may or may not be based on the same information that drives the accuracy of their responses (Macmillan & Creelman, 2004; Pleskac & Busemeyer, 2010; Vickers, 1979; Zylberberg, Barttfeld, & Sigman, 2012). Alternatively, the Appendix tests a simple algorithm which updates a running mean estimate and compares the updated value with its predecessor. If the estimated direction has not changed much, then the observer can be reasonably confident that the direction estimate is accurate, or at least stable. With a criterion on the absolute magnitude of the moment-to-moment change, this scheme accounts for saturating information growth functions consistent with a primacy gradient and later switch times for poor quality information.

In summary, the foraging framework makes the assumption that observers monitor some measure of (un)certainty around the variable of interest (motion direction in this specific instance) and use this to drive their sampling strategy. We sought to test this assumption. We showed that certainty increases with time, up to a plateau. When we experimentally manipulated observers’ (un)certainty, by varying coherence and inserting coherence pulses, we were able to measure the consequences both in terms of the accuracy of direction report (Experiments 1–3) and in terms of their active sampling strategy (Experiment 4). These findings support the idea that some online measure of (un)certainty is computed by observers, but its algorithmic implementation in real time is an open question.

## Figures and Tables

**Table 1 tbl1:** Bayes Factors on the Information Growth Parameters in Experiments 1 and 2

Model	τ	λ
Experiment	.58	1.84
Coherence	.56	3.62 × 10^4^
Experiment + Coherence	.33	2.04 × 10^5^
Experiment × Coherence	.26	8.34 × 10^5^
*Note*. Bayes factors in favor of the model are in the left-hand column against a null-model that contains just a subject-specific intercept.		

**Table 2 tbl2:** Gaze Time Allocation, Switch Frequency and Accuracy of Perceptual Decisions in Experiment 4 (95% Within-Subject Confidence Intervals in Parentheses)

Performance measure	No pulse				Pulse			
	LL	LH	HL	HH	LL	LH	HL	HH
Gaze time	.57 (± .02)	.58 (± .02)	.53 (± .01)	.54 (± .01)	.56 (± .01)	.56 (± .02)	.55 (± .01)	.55 (± .01)
P(1 switch)	.82 (± .03)	.79 (± .03)	.80 (± .03)	.77 (± .06)	.79 (± .04)	.79 (± .02)	.78 (± .05)	.77 (± .04)
Perceptual accuracy	.61 (± .04)	.69 (± .04)	.65 (± .03)	.79 (± .04)	.67 (± .04)	.75 (± .04)	.70 (± .04)	.80 (± .02)
*Note*. The two-letter strings in the table header denote the coherence of the first and second pattern, respectively (L = low; H = high).								

**Figure A1 fig11:**
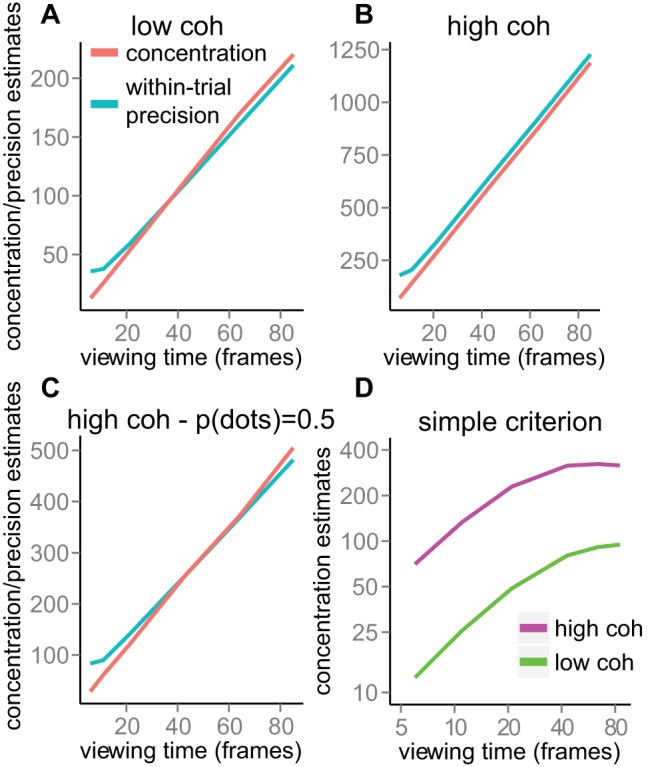
RDK simulation results. A – Low coherence, with all dots taken into account. B – High coherence with all dots taken into account. C – High coherence with only half the dots taken into account. D – Information growth for low and high coherence patterns, with a fixed criterion on the absolute change in the running mean estimated direction. Integration of information stops as soon as the change in the running average drops below the criterion. In this simulation, the criterion was set to 5 × 10^−4^ radians. The resulting direction estimates are fit with Von Mises densities. Here we show that with this mechanism in place, sampling stops prematurely, which leads to asymptotic growth in the concentration of the direction estimates. Note the data are plotted on double-log coordinates to allow both low and high coherence data to be shown in the same panel. See the online article for the color version of this figure.

## Footnotes

1 To see why this is the case, note that the derivative of the growth curve $\frac{d\kappa }{dt}=\frac{\lambda }{\tau }e^{-\frac{t}{\tau }}$. Adopting a criterion of θ, we set $\frac{d\kappa }{dt}=\theta$. Solving for *t* gives $t=-\tau \;\ln\;\frac{\theta \tau }{\lambda }$. As asymptotic concentration, λ, increases, the fraction inside the logarithm approaches 0 (assuming λ > θτ), resulting in increasingly negative numbers that are multiplied by −τ.

## Estimating Within-Trial Precision From Across-Trial Errors

In the main article, we measure the distribution of direction errors to infer how the (un)certainty of the estimated direction changes with time. The foraging model shown in Figure 1 is concerned with the sampling strategy on an individual trial. The model makes the assumption that the decision to switch to a new source of information is governed by some criterion level of precision of the estimated variable (i.e. direction). Therefore, it is important to understand the relation between the information accrual *within an individual trial* and our measure of the concentration of directional errors *across trials* at different time points.

The following analogy helps to clarify our logic. Imagine we want to know how the accuracy with which we can estimate a mean IQ depends on the number of participants we test. Clearly we can estimate this by testing *N* participants, computing the mean IQ and the standard error around that mean, for various values of *N*. An alternative, laborious approach would be to compute the mean IQ separately for *M* independent groups of *N* subjects, drawn randomly from the population of interest (and repeat this for various values of *N*). The standard deviation of the distribution of *M* means provides an estimate of the expected error with which the mean IQ from any one group of *N* participants approximates the population mean.

Now let us apply this logic to the motion direction judgements of Experiments 1 through 3, with a simulation. In these experiments we control viewing time, or the number of samples within a trial (*N*). We think of a ‘sample’ as a global, average direction estimate across a number of dots (not necessarily all of them) across a number of frames. We will refer to these as ‘direction samples.’ As the samples come in, the simulated participant computes a running (circular) mean over all the samples seen so far. In addition, (s)he estimates the (circular) standard error of the mean. We convert this into a precision measure by taking the reciprocal of the squared standard error. As the samples come in, the standard error goes down and the precision increases (approximately linearly once sufficient samples are available). At the end of the viewing epoch, the observer reports the final value of the running mean. Across *M* trials, we have a distribution of errors around the true motion direction. The question is: how does spread of this distribution across trials relate to the average precision within trials?

We performed some simple simulations to assess this relationship. These simulations also addressed the question whether the limited growth in the concentration values is somehow inherent in the informational content of the RDK patterns. The specific assumptions of the simulation were as follows. (a) The signal direction was always 0° (horizontal right). The directions of noise dots were drawn in the same way as for the patterns in our experiments. (b) The observer computes a circular mean of the direction over all or a random sub-set of dots across successive frames. This estimate constitutes one direction sample. (c) After the final frame *N*, the reported direction is the circular mean of all the direction samples from that trial. (d) The precision is computed as the reciprocal of the squared standard error around that mean after *N* frames. (v) After *M* trials (*M* = 10000), the distribution of errors is fit with a Von Mises density. The concentration parameter of that distribution measures the reciprocal spread of that distribution, analogous to the precision around the mean measured on individual trials.

Figure A1A and A1B show the results of this simulation for low and high coherence patterns. We averaged the within-trial precision over *M* trials; these values are shown as ‘within-trial precision.’ These precision estimates are not very well estimated for the shortest viewing duration (*N* = 6), where only 5 direction samples are available. However, for longer viewing times the within-trial precision increases linearly. Importantly, the concentration parameter computed from the across-trial error distributions also increases linearly with viewing time, and is typically very close to the within-trial precision. We think the discrepancy for the short viewing durations is down to a bias in the estimated standard error of the mean on individual trials after 6 frames. However, the concentration parameter for this duration is estimated from a relatively well-defined distribution of errors across trials. Note that the overall concentration values attained in the high coherence condition are much larger than in the low coherence condition. This is consistent with the rate-of-rise effect shown in Figure 2 in the main text.[Fig-anchor fig11]

The concentration values achieved by the simulated observer are much larger than those measured in our human participants. The model we simulate here is clearly not realistic in that it ignores any spatiotemporal filtering by the early visual system and higher-level motion sensitive mechanisms. There are clearly various sources of inefficiency and noise in human participants that are not taken into account here. One example of inefficiency is that humans are unlikely to compute the global direction across all the dots. Figure A1C shows a simulation in which the coherence was high, but the subject samples a random subset of half the dots on every frame (Dakin, Mareschal, & Bex, 2005). Unsurprisingly, this form of inefficiency reduces the overall concentration values that are achieved. Nevertheless, the linearity with viewing time and the close relation between across-trial concentration and within-trial precision, is preserved.

Finally, we simulated the information growth for low and high coherence patterns at various viewing durations, with a criterion on the absolute change in the running mean across two successive direction samples. This comparison was meant to implement a simple heuristic for monitoring uncertainty: if a new direction sample does not alter the estimated direction very much, then this estimated direction is likely to be relatively stable. One way to think of this algorithm is in terms of a predictive coding mechanism. That is, the observer uses the samples observed so far to generate a prediction about a new, incoming sample. If that sample is highly consistent with the prediction (small prediction error), then arguably the direction estimate is sufficiently accurate. If the incoming sample is inconsistent with the prediction and would result in a change in the mean direction estimate, then it is worth continuing to sample more information.

The consequence of this simple heuristic is that frequently—particularly for the longer viewing times—the observer has already decided on the motion direction before the viewing period is over (cf. Ratcliff, 2006). The later direction samples are ignored and the reported direction is whatever the estimated mean direction was at the time when |*m*_*t*_ − *m*_*t*−1_| ≤ θ, where *m* is the mean of all the direction samples observed until the time indicated in the subscript. In this simulation θ = 5 × 10^−4^ radians. Figure A1D shows that this simple mechanism can account for the saturating concentration values for the longer viewing durations, which is a consequence of a primacy gradient. In addition, this mechanism—if used to determine a switch point in the comparative task—predicts that a high coherence pattern will be left sooner than a low coherence pattern, with a single criterion in place.
